# Factors Influencing Adherence to the Risk Management Program for Women With a Genetic Predisposition to Breast Cancer: Real-World Data from a French Multicenter Program

**DOI:** 10.1093/oncolo/oyae057

**Published:** 2024-04-02

**Authors:** Ke Zhou, Martine Bellanger, Louise Crivelli, Sandy Laham, Charlotte Huet, Caroline Abadie

**Affiliations:** Department of Human and Social Sciences, Institut de Cancérologie de l’Ouest (ICO), Saint-Herblain, France; Department of Human and Social Sciences, Institut de Cancérologie de l’Ouest (ICO), Saint-Herblain, France; Department of Health Promotion and Prevention, Institut de Cancérologie de l’Ouest (ICO), Saint-Herblain, France; Department of Oncogenetics, Eugène Marquis Cancer Center, Rennes, France; Department of Health Promotion and Prevention, Institut de Cancérologie de l’Ouest (ICO), Saint-Herblain, France; Coordination of the Phare Grand Ouest Program, Eugène Marquis Cancer Center, Rennes, France; Department of Human and Social Sciences, Institut de Cancérologie de l’Ouest (ICO), Saint-Herblain, France; Department of Oncogenetics, Institut de Cancérologie de l’Ouest (ICO), Saint-Herblain, France

**Keywords:** *BRCA1*, *BRCA2*, oncogenetics, breast cancer, screening, risk management program, prevention

## Abstract

**Background:**

Risk management programs targeting women with genetic predispositions to breast cancer (BC), eg, *BRCA1* and *BRCA2*, are effective assuming full adherence with the program protocol. However, high risk to BC in women and equal access to care may not result in high and uniform adherence with the program.

**Objective:**

To elucidate factors influencing adherence with screening program in women with genetic predispositions to BC.

**Material and Methods:**

We retrieved data from a multicenter pathogenic-related BC surveillance program across 4 French regions. We used multilevel logistic modeling to analyze factors of adherence with the program, with “on-time” or postponed screening as the dependent variable.

**Results:**

Seven hundred and seventy-eight participants were followed for a 4.7-year median. We observed 2796 annual screening rounds and 5.4% postponed rounds with a 6-month margin. Women with prevalent BC and carriers of *BRCA1* and *BRCA2* mutations did not have on-time annual screenings any more than women low cancer risk. Better adherence was observed with screenings after the 2nd round, with higher total number of rounds. Having one or more recalls was significantly associated with worse adherence. No contextual factors affected adherence. Furthermore, postponed rounds increased between 2018 and 2020 compared to 2015 and 2017.

**Conclusion:**

Having a higher BC risk status does not result in better adherence to the risk management program. However, factors directly related to screening rounds reduced postponements. Future research should address the benefits of screening-related organizational factors that contribute to adherence improvement.

## Teaser

This article reports factors influencing adherence with screening program protocol in women with genetic predispositions to breast cancer.

**Implications for Practice**

Women with a genetic predisposition to breast cancer (BC) have a markedly increased lifetime risk of developing the disease. Despite their awareness, they do not achieve 100% adherence to screening in BC risk management programs. This study found that the most important factors in women’s adherence are those directly related to screening rounds, rather than those related to women’s risk profile or contextual factors. This implies that strategies to improve adherence should target screening rounds, for instance by sending reminders, creating interactions between the screening program team and participants, and using other organizational facilitators.

## Background

Approximately 5% to 10% of breast cancer (BC) cases can be associated with a genetic predisposition,^1^ among which *BRCA1* and *BRCA*2 are the most commonly involved genes. These pathogenic gene mutations confer high lifetime risk of BC as reported in 2 metanalyses.^2,3^ This was confirmed by findings from a recent, large international prospective cohort study where women were recruited “on the basis of their mutation status and followed over time.”^4^ Women carrying the *BRCA1* pathogenic variant had an estimated mean cumulative risk of 72% (95% CI: 65%-79%), while women carrying the *BRCA*2 pathogenic variant had a mean risk of 69% (95% CI: 61%-77%) to develop BC by the age of 80.^4^ Women with these mutations often develop BC at younger ages, with increased incidence primarily starting at 30 years old.^4^

BC risk management interventions targeting germline *BRCA1* and *BRCA*2 pathogenic variant carriers include intensive BC screening for early detection and risk-reducing surgery (ie, prophylactic mastectomy).^5-10^ On one hand, screening using magnetic resonance imaging (MRI) shows high sensitivity and specificity in high-risk populations.^8,9^ On the other hand, it is essential to ensure that women attend their screening appointments regularly and punctually. Women’s adherence to screening is the most crucial factor of effectiveness of the screening program.^6^ In the general population, BC screening often faces suboptimal adherence.^11-20^ Efforts to optimize participant adherence using behavioral change techniques have been mostly unsuccessful.^12^ However, simple and direct invitation techniques such as sending reminders and prompts, fixing appointments, and organizational improvements had more positive direct effects than complex interventions, such as counseling or home visits.^13^

The actual evidence on whether women’s higher risk of BC would increase adherence to screening is limited.^6^ In women diagnosed with primary BC, a Dutch study reported that even women with a higher risk of recurrence did not increase their adherence to secondary prevention surveillance.^21^ An American study, likewise reported that high lifetime risk (ie, above 40%) in women with equal healthcare access, due to their military status, was not associated with uniform high adherence to screening.^22^ To the best of our knowledge, adherence analysis based on high-risk status is not available in France.

The present article aims to fill the gap and contributes to a better understanding of the main factors that influence adherence to screening for women with a genetic predisposition to BC. To investigate the factors of adherence to the surveillance guidelines, we model real-world data collected from 8 cancer centers in a large region of western France.

## Material and Methods

### Study Setting

The Phare Grand Ouest program, called PGO hereafter, is a surveillance management program for individuals at high risk of pathogenic-related cancers including BC. PGO consists of 8 oncogenetics teams and a coordination team at an interregional level, which serves 4 western French regions with approximately 11 million inhabitants. Women included in PGO have the *BRCA1* or *BRCA*2 pathogenic variant without prophylactic bilateral mastectomy and prophylactic salpingo-oophorectomy and without progressive cancer. PGO is also offered to high-risk BC women without the *BRCA* pathogenic variant, defined by an estimated lifetime BC risk >20%-25% calculated with the Breast and Ovarian Analysis of Disease Incidence and Carrier Estimation Algorithm—Boadicea-model.^23,24^ Since PGO was initiated in 2011, the Boadicea versions used did not include non-familial BC risk factors such as breast density, reproductive, and lifestyle patterns.

Prior to being included in PGO, women with newly diagnosed breast or ovarian cancer meeting the criteria for genetic predisposition based on age at diagnosis and/or familial history, so called probands or *index cases*, were referred to specialized genetic services, which conduct detailed 3-generation family tree analysis and a germline testing of genes involved in hereditary breast and ovarian cancer (HBOC). PGO participation is offered to women carrying a germline *BRCA1* or *BRCA*2 pathogenic variant. Their relatives then undergo a predictive targeted genetic test with carriers also being allowed to participate in the PGO program. In the absence of known pathogenic variants identified in *index cases* genetic consultations determine the level of BC risk. Only women considered at high risk among those without known mutations benefit from PGO surveillance. Specific consent is obtained throughout the genetic consultations, during which the results are shared with the woman after assessing her BC risk level. The PGO program is only offered to women with a genetic high risk, either with a personal history of BC or cancer-free women. We assume that most women with a personal history of BC were “index cases” and most cancer-free women were relatives.

### Surveillance protocol

PGO manages the high-risk BC surveillance with annual postal mailings to women 3-months prior to their scheduled screening along with several reminders when follow-up examinations are not reported. The initial mailing is a reminder for women to schedule their appointments at the radiology clinic of their choice. Additional postal mailing and/or phone reminders are made when necessary (ie, at 2 month, 4 or more-month delays). PGO provides guidance on the recommended BC screening along with updated follow-up recommendations.

In France, for cancer-free women aged 30 to 65 years, the surveillance protocol consists of an annual MRI screening supplemented with mammography (one single-incidence per breast). Cancer-free women over 65 only receive a mammography (2 incidences per breast), without an MRI. Women with a history of cancer receive an annual MRI supplemented by mammography (2 incidences per breast) for women 30 to 65 years; women over 65 an annual mammography (2 incidences per breast), with no MRI.^25^ The schedule scheme is based on MRI and mammogram performed as much as possible at the same time or otherwise without exceeding 2 months. Additionally, PGO shares a personalized follow-up plan with women and their general practitioners (GPs) or specialists such as radiologists, gynecologists, and oncologists. In some radiology clinics, the personalized follow-up plan serves as a prescription.

### Study Population

The study population consisted of women with a signed PGO consent form and without bilateral mastectomy, prophylactic or curative, before the surveillance program started, and had at least one screening round between January 01, 2015 and March 17, 2020. The latter was the start of the nationwide lockdown in France during which the restrictions impacted access to imaging in clinical settings (Fig. 2).

### Outcome of Interest and Explanatory Variables

**Figure 1.**
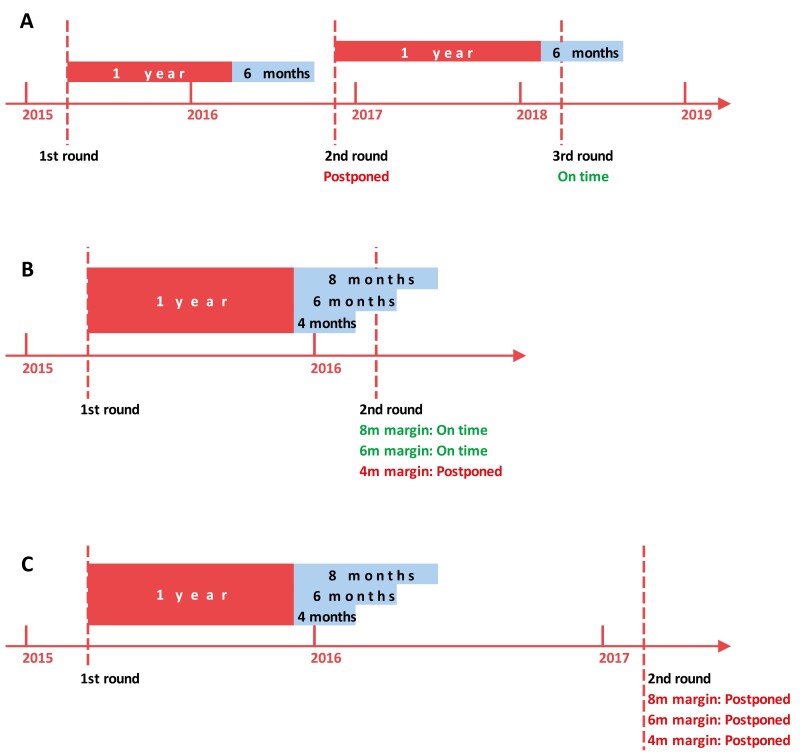
Definition of postponed screening rounds. (**A**) The 2nd round occurs beyond one year plus a 6-month margin and is therefore a postponed round. The 3rd round take place within 1 year plus a 6-month margin from the date of the 2nd round, making it an on-time screening round. (**B**) Whether or not the 2nd round is postponed depends on the chosen margin. (**C**) The 2nd round occurs beyond 1 year plus a 8-month margin, and is therefore a postponed round, regardless of the chosen margin.

The outcome of interest is the extent to which women were punctual with screening protocol within a tolerance margin of delay, defined by expert consensus. As illustrated in Fig. 1, for round *n*, we calculated the time interval between *n* and (*n* − 1) rounds to determine whether round n was postponed. A round was considered postponed if an MRI had an interval of 18 months (12 + 6 months) or more from the previous round; otherwise, the screening was considered on-time.

As explanatory variables, we noted the number of screenings, with the first round being the first year of screening, and the 2nd round being the 2nd year, until the end of the observation period. We included the year of each round to account for changes in outcomes over time.

We used the variable ‘birth cohort’ to denote the women’s birth years, which remains constant and further calculated their age at each screening. We estimated the total number of past recall exams (ie, previous imaging with suspicious results) at each screening along with the total number of rounds during the observation period. We included *BRCA1* and *BRCA*2 mutation status and high risk of BC without mutation as explanatory variables for women. In addition, we included prevalence BC status, ie, with or without cancer development before the women were included in PGO. Of note, prevalent BC means any history of BC for which treatment was completed prior to PGO inclusion.

We collected the following contextual variables: Standard of living at municipality level,^26^ the Local Potential Accessibility (LPA) indicator, which measures accessibility to private GPs, standardized by age structure within municipalities,^27^ and MRI density measured as the number of MRI screenings given per 200 000 inhabitants in the county of residence.^28^ To create binary variables, we used the median value as the cutoff value for contextual data.

### Statistical Analysis

We reported mean, range, and standard deviation (SD) of symmetric distributed variables, and median and interquartile ranges IQR (25th and 75th percentiles) for variables with skewed distributions. When relevant, we used a bootstrap sampling technique with 1000 replications to estimate 95% confidence intervals (95%CI). We used the Kruskal-Wallis test and Fisher’s exact test to determine the difference between subgroups for continuous variables and count data, respectively.

In the univariable analysis, we investigated whether a single explanatory variable was associated with our outcome of interest noted (0) for an on-time and (1) for a postponed round.

In the multivariable analysis, we used a generalized linear model (GLM) with a logit link function to estimate the odds ratios (ORs) of woman’s adherence to the surveillance program. We used a multilevel modeling approach that accommodated the structure of our data. In other words, data on screening rounds (level 1) were nested within women (level 2). The latter are usually nested within a care setting (as level 3). However, in our study, women included by the 8 oncogenetic teams are free to choose their screening/imaging setting, which is not necessarily the cancer centers or hospitals from their inclusion. Therefore, after further tests described below in the modeling process, we limited our models to the first 2 levels.

We built the models, starting with an empty one with no random effect (model 0) and then added levels of analysis.^29^ We tested the benefit of adding a level using the Bayesian Information Criterion (BIC).^30^ To estimate how much outcome differences were distributed between random effect units, we obtained the empty random effect model (model 1) without any explanatory variables and used the variance partition coefficient (VPC) to assess how much of the total variation in women’s adherence was explained by the variation among women at level 2.^31^ Performing the same procedures for cancer centers did not improve model fit and variation in outcome means estimated between centers was too negligible to consider a center as having a higher level of random effect. To model some relationships of interest, we built an intermediate model (model 2) by including screening round related explanatory variables into model 1 and a full model (model 3) by adding women’s characteristics and contextual factors (at women level).^29^ Last, we performed a sensitivity analysis by extending the tolerance margin from 6 months to 4 and 8 months to estimate the effects on postponed screenings. All statistical analyses were performed using the STATA 14.2 package.

## Results

**Figure 2.**
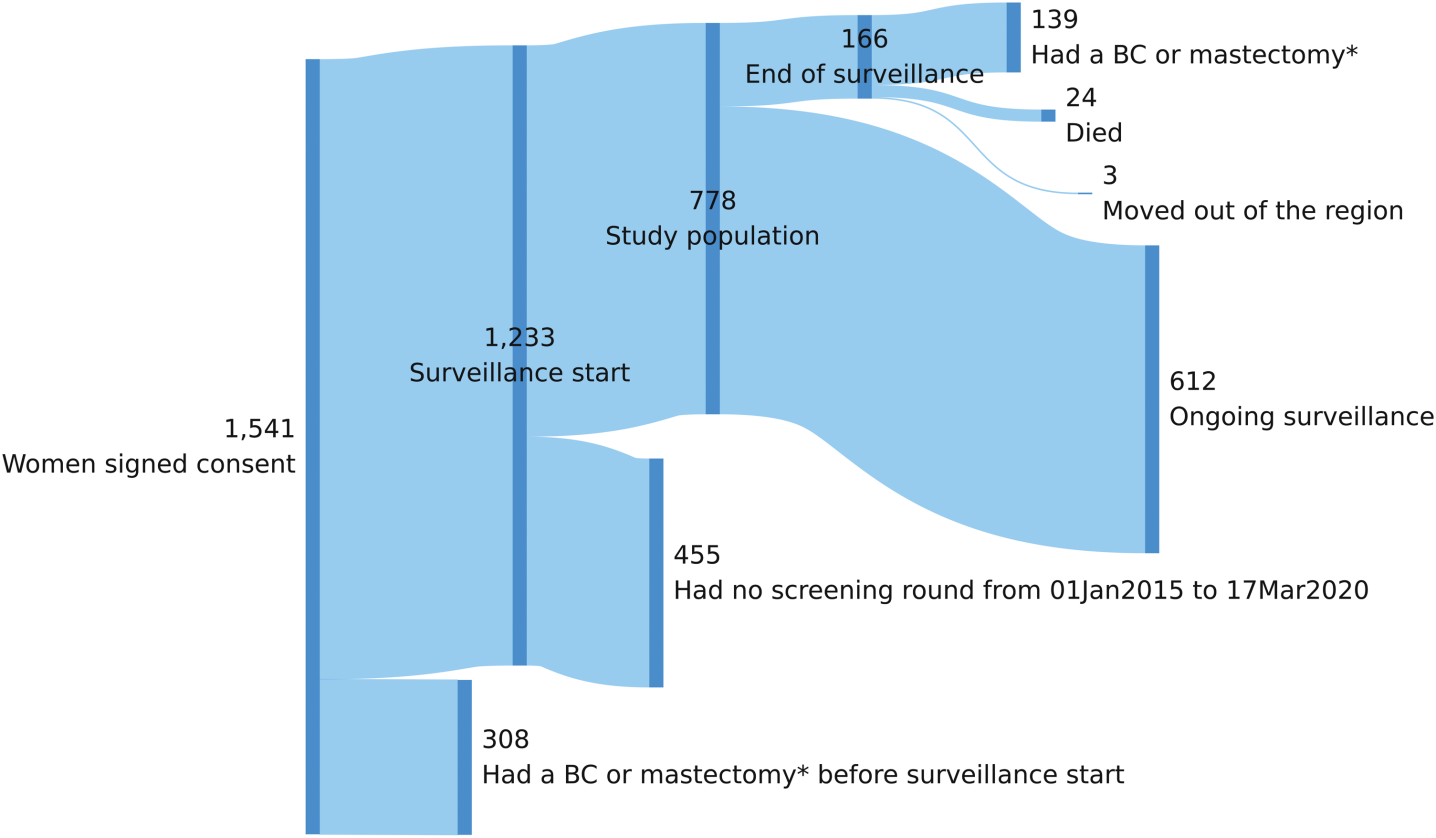
Sankey flow diagram of PGO study population. *BC, breast cancer; Mastectomy—bilateral mastectomy, prophylactic, or curative.

Between 01 January 2015 and 17 March 2020, 778 women participated in the PGO program and underwent at least one screening round. Of these, 612 (79%) were under active surveillance until 17 March 2020 (Fig. 2). Median age at inclusion was 45.2 (Table 1). Nearly half of the women had prevalent BC (344/778), and were older (p < 0.001) than those without it (median age at inclusion 49.3, IQR 43.1-55.0, *n* = 344; versus median age at inclusion 41.5, IQR 34.9-48.2, *n* = 434, respectively). Overall, women had a median of 0.96 rounds per year (IQR: 0.77 to 0.99, 95%CI 0.95-0.96). The median interval between 2 rounds increased from 371 days in 2016 (IQR: 357-385 days, 95%CI: 368-374 days) to 377 days in 2019 (IQR: 358-406 days, 95%CI: 373-381 days).

**Table 1.** Patient characteristics and contextual factors.

	*n* (%)
Total	778 (100)
Age at incl. (years)	
Median (IQR)	45.2 (37.8-52.0)
Under 40	256 (33)
40-49	276 (35)
50 and over	246 (32)
Mutation status	
* BRCA*1	287 (37)
* BRCA*2	218 (28)
H. risk w/o *BRCA*	273 (35)
Prevalent BC	
No	434 (56)
Yes	344 (44)
Standard of living at municipality level (€)	
Median(IQR)	20 479 (19 510-21 810)
less than 20 000	288 (37)
20 000-24 999	473 (61)
25 000 and more	17 (2)
Local potential accessibility (LPA)	
Median(IQR)	3.7(3.1-4.4)
less than 2	24 (3)
2 to 3.9	457 (59)
4 to 5.9	272 (35)
6 and more	25 (3)
No. of MRI/200 k hab. of the county of residence	
Median(IQR)	1.9(1.7-2.4)
less than 1	115 (15)
1 to 2.4	506 (65)
2.5 to 3.9	113 (15)
4 and more	44 (6)

Overall, we observed a total number of 2796 rounds (3.4 rounds/woman) with 5.4% of them being postponed screenings with a 6-month margin (Table 2). In univariable analysis, order of rounds, year of rounds, and total number of rounds had significant effects on women’s adherence, regardless of the tolerance margin. In addition, women’s age at a screening round and number of past recall exams also had significant effects on adherence (Supplementary Table S1).

**Table 2.** Women’s adherence with PGO, by mutation status and prevalent BC status.

	*BRCA*1	*BRCA*2	H. risk w/o *BRCA* 1 or 2	Total
	*n* (%)	*n* (%)	*n* (%)	*n* (%)
No. of women without prev. BC	164	131	139	434
Observation duration (years), median (IQR)	4.7(2.0-5.1)	4.7(3.7-5.1)	3.9(3.4-4.7)	4.3(3.4-5.1)
Total no. of screening rounds performed	553(100)	467(100)	477(100)	1497(100)
No. of postponed screening rounds				
4 months margin	49(9)	44(9)	52(11)	145(10)
6 months margin	25(5)	28(6)	31(6)	84(6)
8 months margin	18(3)	14(3)	15(3)	47(3)
No. of women with prev. BC	123	87	134	344
Observation duration (years), median (IQR)	4.7(3.1-5.1)	5.1(5.1-5.1)	5.0(4.5-5.2)	4.8(4.5-5.1)
Total no. of screening rounds performed	464(100)	323(100)	512(100)	1299(100)
No. of postponed screening rounds				
4 months margin	33(7)	16(5)	29(6)	78(6)
6 months margin	20(4)	14(4)	19(4)	53(4)
8 months margin	13(3)	8(2)	11(2)	32(2)
Total no. of women	287	218	273	778
Observation duration (years), median (IQR)	4.7(3.1-5.1)	4.9(3.7-5.1)	4.7(3.4-5.0)	4.7(3.4-5.1)
Total no. of screening rounds performed	1017	790	989	2796
No. of postponed screening rounds				
4 months margin	82(8)	60(8)	81(8)	223(8)
6 months margin	45(4)	42(5)	50(5)	137(5)
8 months margin	31(3)	22(3)	26(3)	79(3)

**Figure 3.**
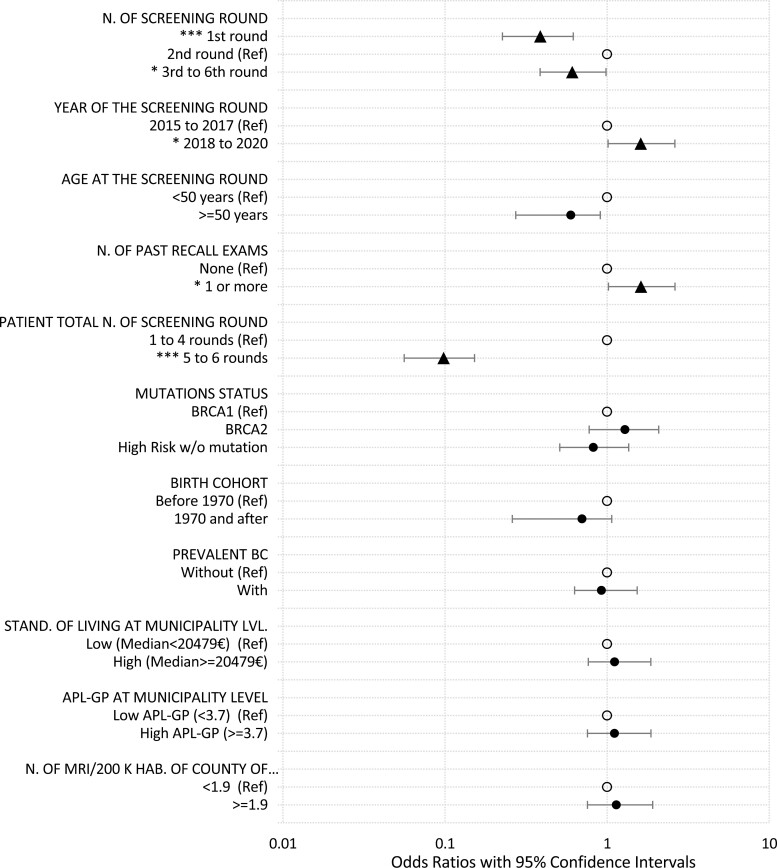
Results of multivariable analysis (6 months margin, full model). Significant levels: ***P-value < .001; **P-value < .01; *P-value < .05; red spots: odds ratios significantly difference from 1; black spots: odds ratios non-significantly difference from 1; empty spots: reference categories.

Figure 3 shows the multivariable analysis of the effects of factors on women’s adherence to the PGO program. After adjusting for other covariables, the odds of postponements in round 1, round 3, and subsequent rounds were 0.39 (95%CI: 0.23 to 0.65) and 0.61 (95%CI: 0.38 to 0.99) times lower than in round 2, respectively (Supplementary Table S2, model 4). Better adherence was also observed when a woman had more than 4 total rounds. In contrast, we observed no significant differences in adherence across prevalent BC status. Similarly, mutation status did not show a significant effect on adherence. The odds of postponed rounds increased by 0.61 (odds ratio 1.61, 95%CI: 1.01 to 2.59, 1.61 to 1.00 = 0.61) between 2018 and 2020 relative to those between 2015 and 2017. Surprisingly, having undergone one or more recall exams prior to a round was not associated with better adherence, but significantly increased the odds of postponements by 0.62 (odds ratio: 1.62, 95%CI: 1.00 to 2.61, 1.62 to 1.00 = 0.62). The “contextual factor” such as standard of living, availability of GPs and MRI density in the living area, had no effect on adherence (Supplementary Table S2, model 4).

## Discussion

In this study, we investigated factors for women’s adherence to screening protocols in a French risk management program for women at high risk of pathogenic-related BC. We found that women with prevalent BC did not attend annual screenings on time any more than BC-free women. The variability in round postponement can be explained by screening factors such as: order of rounds, year and age at a round and total number of rounds. Women’s characteristics (pathogenic variant status, birth cohort, or prevalent BC status) as well as contextual factors (standard of living, accessibility to GP, MRI density) do not explain variability in women’s adherence to the program.

Although the surveillance protocol is set for one year, the women took more than a year to attend the following round. Consequently, more than 5% of the rounds were postponed with a 6-month margin. The screening factors had a significant influence on adherence. Women at the first round of screenings had rarely postponed exams. Among women who had the first round of screenings, exams were rarely postponed. The proportion of postponed exams increased by the second round, and then decreased for the third and subsequent rounds. This is likely because women receive more direct aid and instructions for an MRI exam during the first round, however, starting from the third round and on, they became more autonomous leading to an increase in adherence. Consistently, women with more total rounds had fewer postponed screenings overall due to their acquired habit of undergoing exams over the years. This highlights the importance of organizational facilitators for adherence.^13^

In terms of adherence to the surveillance program, our multivariable model showed that mutation status and prevalent BC status had no effect on reducing postponed rounds. This is consistent with the conclusion of Draeger and colleagues’ study in the Netherlands where higher risk of BC recurrence in women was not associated with better adherence to secondary prevention surveillance.^21^ Similar to Rollet et al’s French national BC screening program, our univariable analysis confirms an increase of adherence with age. Having previous suspicious screening results that led to recall exams had a significant negative effect on adherence to the program. The latter effect might be associated with women’s increased anxiety due to additional exams that might influence their acceptance of subsequent screenings and lead to delayed attendance, despite the personalized follow-up offered by PGO. Additionally, we identified a confounding factor in the relationship between age, BC prevalence, and screening delay. Indeed, BC prevalence and advanced age resulted in significantly fewer screening postponements in the univariable analysis, which became non-significant after adjustment in the multivariable model.

Population-based BC screening assumes that women have an average cancer risk. Their adherence to national screening programs depends largely on contextual factors such as socioeconomic status and geographical access.^14-17^ However, this conclusion was inconsistent across studies.^13,14^ Interestingly, our study suggests that the disparity in contextual factors did not explain the variability in postponement of single rounds. Cautiously interpreted, women with a lower standard of living or worse access to healthcare services may not have lower screening uptake, underscoring the value of a specific risk management program to reduce screening inequalities. In addition, this reflects, to some extent, the findings of Do and colleagues where equal access to healthcare did not result in high and uniform adherence to the risk management program.^22^ However, when examining the rounds with a broader scope of 3-year intervals, we observed an overall increase in time between 2 rounds, which might be related to contextual factors unobserved in our study. Among these factors, increased demand for MRI, while most OECD (Organization for Economic Co-operation and Development) countries, France included,^32^ have faced recently a shortage of radiologists, and an increase in the workload, has likely resulted in longer access delay.

Overall, our study shows high participant adherence to PGO, the proposed surveillance and BC risk management program. This finding may be associated with their limited financial contribution. The program is part of the national health insurance (NHI) coverage, meaning that annual screening and related procedures are reimbursed by the NHI, and additional costs are covered by voluntary complementary insurance schemes. However, globally not all healthcare systems provide this benefit to women at high risk for BC. This should be considered carefully before generalizing our findings.

One of the limitations of our study is that we used aggregated data on living standard, accessibility to GP estimates at the municipality level, and MRI density estimate at the county level. We acknowledge that the association between individual and “area-level” measures used might not be fully consistent. Additionally, available data did not enable us to use additional socioeconomic factors such as education level, employment/occupation indicators, and social disparity indicator such as the European Deprivation Index (EDI).^14^ Similarly, we were not able to assess the distance and travel time of women to MRI facilities since their exact location was missing. Last, the paucity of the available data hampers accurate estimation. Our study did not assess the association between the screening results (BI-RADS) and participants’ feedbacks such as patient-reported outcomes (PROs) and their adherence to PGO.

The strength of our study lies in investigating screening adherence in women at high risk of BC with different approaches. First, we used informative outcomes to demonstrate simple differences or no difference in screening program adherence between women with different characteristics. We then used a multilevel regression model to investigate factors in 3 different categories (screening, women, and contextual) related to adherence. We applied a postponement tolerance margin, which increased the robustness of our results. Previous studies showed that compared to complex behavioral change interventions to improve BC screening adherence, organizational facilitators are more effective and cost-effective.^6,13^ However, no screening adherence study has been done for women with a genetically high risk for BC. We might assume that in our study population, where the perception of BC risk is likely increased, screening adherence would be influenced by an individual’s risk profile. However, our findings show that adherence to screening is primarily influenced by the factors related to screening organization, rather than individual risk profile.

## Conclusion

PGO is a program that constantly leads to adjustments in the personalized screening plan for each round, creating interactions between the screening program team and the participants, by sending reminders and conducting phone calls, to ensure that women’s screening patterns are consistent with their risk profile and national recommendations (22). In the case of an abnormal imaging result, the program coordinator offers organizational facilitators such as personalized reminders in order to fit with an optimal BC risk management protocol.

Analyzing the adherence factors of the risk management program in women with high risk of BC is of particular importance. Our results showed that factors directly related to screening rounds help reduce postponements.

In light of the observed effects of screening factors on adherence to the risk management program, our study paves the way to further research which of these organizational factors improve the on-time completion of rounds and on quantifying the benefits of such improvement, such as the reduction in interval cancers.

## Supplementary Material

oyae057_suppl_Supplementary_Tables

## Data Availability

The data underlying this article will be shared on reasonable request to the corresponding author.

## Authorization for Health Data Use

This study does not involve human participants in accordance with the Reference Methodology (ie, MR-004), set up by the French National Data Protection Authority (CNIL). The MR-004 framework applies to “research that does not involve human subjects, studies or evaluations in the health realm.” This applies in particular to studies, which reuse health-related data for research. Inclusion in PGO is part of a prevention program, which in turn falls under the national cancer strategy within the framework of oncogenetics. The Data Protection Officer (DPO) and the CREDO (Comité Recherche et Exploitation des DOnnées) of the data center (Eugene Marquis Cancer Center) gave an agreement on April 28, 2022 for the reuse of the anonymized data for research and scientific journal submission.

## Patient and Public Involvement

Patients and/or the public were not involved in the design, conducting, reporting, or dissemination plans of this research.
