# Supplementary material for: Factors Influencing Adherence to the Risk Management Program for Women With a Genetic Predisposition to Breast Cancer: Real-World Data from a French Multicenter Program
Source: Oncologist. 2024 Apr 2;29(8):e967–75. doi: 10.1093/oncolo/oyae057 (PMC11299934; doi:10.1093/oncolo/oyae057)
Supplement: oyae057_suppl_Supplementary_Tables [file oyae057_suppl_supplementary_tables.pdf]

Supplementary Tables

Factors Influencing Adherence to the Risk Management Program for Women with a Genetic Predisposition to Breast Cancer: Real-World Data from a French Multicenter Program

Supplementary Table 1. Univariable analysis

1 observation = 1 MRI screening round		Outcome: Postponed screening rounds					
		Margin = 4 mths		Margin = 6 mths		Margin = 8 mths	
Covariables	N	n (%)	p-value	n (%)	p-value	n (%)	p-value
N of screening round			<0.01		<0.001		<0.001
1st round	750	63 (8)		26 (3)		7 (1)	
2nd round	688	73 (11)		56 (8)		37 (5)	
3rd to 6th rounds	1358	88 (6)		56 (4)		36 (3)	
Year of the screening round			<0.001		<0.001		<0.001
2015 to 2017	1564	51 (3)		38 (2)		21 (1)	
2018 to 2020	1232	110 (9)		74 (6)		52 (4)	
Age at the screening round			<0.05		<0.01		0.06
<50 years	1525	102 (7)		76 (5)		48 (3)	
>=50 years	1271	59 (5)		36 (3)		25 (2)	
Number of past recall exams**			<0.01		<0.01		0.11
None	2336	120 (5)		82 (4)		56 (2)	
1 or more	460	41 (9)		30 (7)		17 (4)	
Patient Total N of screening rounds			<0.001		<0.001		<0.001
1 to 4 rounds	1343	187 (14)		124 (9)		73 (5)	
5 to 6 rounds	1453	37 (3)		14 (1)		7 (0)	
Pathogenic variant			0.68		0.62		0.911
BRCA1	1017	59 (6)		36 (4)		28 (3)	
BRCA2	790	41 (5)		33 (4)		19 (2)	
V. High Risk w/o *BRCA1* or *2*	989	61 (6)		43 (4)		26 (3)	
Birth cohort			<0.01		<0.01		<0.05
Before 1970	1545	72 (5)		46 (3)		31 (2)	
1970-1989	1251	89 (7)		66 (5)		42 (3)	
Prevalent BC status			<0.01		<0.05		0.29
Without	1497	103 (7)		72 (5)		44 (3)	
With	1299	58 (4)		40 (3)		29 (2)	
Standard of living of the town/city			0.29		0.29		0.56
Low (<20479€)	1440	76 (5)		52 (4)		35 (2)	
High (>=20479€)	1356	85 (6)		60 (4)		38 (3)	
APL-GP of the town/city			0.57		0.44		<0.05
Low APL-GP (<3.7)	1455	80 (5)		54 (4)		29 (2)	
High APL-GP (>=3.7)	1341	81 (6)		58 (4)		44 (3)	
N of MRI/200 k hab. in the county			0.06		0.05		0.29
<1.9	1485	74 (5)		49 (3)		34 (2)	
>=1.9	1311	87 (7)		63 (5)		39 (3)	

Supplementary Table 2. Mixed-effect models

	6 months margin			
	Model 0: Empty model	Model 1: Empty random effect model	Model 2: Intermediate modal	**Model 3: Full model**
Level 1 - screening rounds, OR (95% CI)				
N of screening round	-	-	***	*******
1st round	-	-	0.421 (0.248 to 0.716)***	**0.387 (0.231 to 0.648)*****
2nd round	-	-	Ref	**Ref**
3rd to 6th rounds	-	-	0.315 (0.189 to 0.525)***	**0.609 (0.375 to 0.989)***
Year of the screening round	-	-	***	*****
2015 to 2017	-	-	Ref	**Ref**
2018 to 2020	-	-	2.590 (1.577 to 4.252)***	**1.612 (1.005 to 2.586)***
Age at the screening round	-	-		
<50 years	-	-	Ref	**Ref**
>=50 years	-	-	0.623 (0.304 to 1.278)	**0.597 (0.310 to 1.150)**
Number of past recall exams	-	-	*	*****
None	-	-	Ref	**Ref**
1 or more	-	-	1.663 (1.006 to 2.750)*	**1.619 (1.003 to 2.613)***
Level 2 - Women				
Fixed effect, OR (95% CI)				
Patient Total N of screening rounds	-	-	-	*******
1 to 4 rounds	-	-	-	**Ref**
5 to 6 rounds	-	-	-	**0.098 (0.054 to 0.177)*****
Mutation status	-	-	-	
BRCA1	-	-	-	**Ref**
BRCA2	-	-	-	**1.289 (0.791 to 2.101)**
V. High Risk w/o *BRCA1* or *2*	-	-	-	**0.863 (0.536 to 1.388)**
Birth cohort	-	-	-	
Before 1970	-	-	-	**Ref**
1970-1989	-	-	-	**0.700 (0.367 to 1.320)**
Prevalent BC status	-	-	-	
Without	-	-	-	**Ref**
With	-	-	-	**0.923 (0.609 to 1.397)**
Standard of living of the town/city	-	-	-	
Low (Median<20479€)	-	-	-	**Ref**
High (Median>=20479€)	-	-	-	**1.112 (0.749 to 1.652)**
APL-GP of the town/city	-	-	-	
Low APL-GP (<3.7)	-	-	-	**Ref**
High APL-GP (>=3.7)	-	-	-	**1.11 (0.753 to 1.636)**
N of MRI/200 k hab. in the county	-	-	-	
<1.9	-	-	-	**Ref**
>=1.9	-	-	-	**1.14 (0.770 to 1.689)**
Random effect				
Variance of random intercept, (95% CI)	-	0.582 (0.085 to 4.001)	0.614 (0.105 to 3.601)	**0.039 (0.039 to 0.039)**
Random slope	-		Age at screening round, centered	
Variance of Random slope, (95% CI)	-	0.006 (0.002 to 0.020)	0.007 (0.002 to 0.244)	**0.005 (0.005 to 0.005)**
VPC of Random effect	-	0.150 (0.025 to 0.549)	0.160 (0.031 to 0.523)	**0.012 (0.012 to 0.012)**
Bayesian Information Criterion (BIC)	1107.409	1105.999	1105.943	**1081.635**

Supplementary Table 3. Sensitivity analysis (4 months margin)

	4 months margin			
	Model 0: Empty model	Model 1: Empty random effect model	Model 2: Intermediate modal	Model 3: Full model
Level 1 - screening rounds, OR (95% CI)				
N of screening round	-	-	***	
1st round	-	-	0.866 (0.580 to 1.293)	**0.790 (0.533 to 1.171)**
2nd round	-	-	Ref	**Ref**
3rd to 6th rounds	-	-	0.392 (0.251 to 0.613)***	**0.667 (0.435 to 1.022)**
Year of the screening round	-	-	***	*
2015 to 2017	-	-	Ref	**Ref**
2018 to 2020	-	-	2.444 (1.600 to 3.744)***	**1.554 (1.034 to 2.333)***
Age at the screening round	-	-	*	
<50 years	-	-	Ref	**Ref**
>=50 years	-	-	0.566 (0.323 to 0.990)*	**0.785 (0.411 to 1.503)**
Number of past recall exams	-	-		
None	-	-	Ref	**Ref**
1 or more	-	-	1.463 (0.926 to 2.312)	**1.507 (0.985 to 2.305)**
Level 2 - Women				
Fixed effect, OR (95% CI)				
Patient Total N of screening rounds	-	-	-	***
1 to 4 rounds	-	-	-	**Ref**
5 to 6 rounds	-	-	-	**0.165 (0.108 to 0.253)***
Mutation status	-	-	-	
BRCA1	-	-	-	**Ref**
BRCA2	-	-	-	**0.968 (0.632 to 1.482)**
V. High Risk w/o *BRCA1* or *2*	-	-	-	**0.800 (0.535 to 1.195)**
Birth cohort	-	-	-	
Before 1970	-	-	-	**Ref**
1970-1989	-	-	-	**0.984 (0.535 to 1.811)**
Prevalent BC status	-	-	-	
Without	-	-	-	**Ref**
With	-	-	-	**0.698 (0.479 to 1.016)**
Standard of living of the town/city	-	-	-	
Low (Median<20479€)	-	-	-	**Ref**
High (Median>=20479€)	-	-	-	**0.945 (0.671 to 1.331)**
APL-GP of the town/city	-	-	-	
Low APL-GP (<3.7)	-	-	-	**Ref**
High APL-GP (>=3.7)	-	-	-	**0.979 (0.700 to 1.369)**
N of MRI/200 k hab. in the county	-	-	-	
<1.9	-	-	-	**Ref**
>=1.9	-	-	-	**1.224 (0.974 to 1.715)**
Random effect				
Variance of random intercept, (95% CI)	-	1.307 (0.637 to 2.681)	1.101 (0.496 to 2.445)	0.544 (0.147 to 2.014)
Random slope	-	Age at screening round, centered		
Variance of Random slope, (95% CI)	-	0.006 (0.001 to 0.023)	0.007 (0.002 to 0.025)	0.003 (0.000 to 0.029)
VPC of Random effect	-	0.284 (0.162 to 0.449)	0.251 (0.131 to 0.426)	0.142 (0.043 to 0.380)
Bayesian Information Criterion (BIC)	1542.179	1551.468	1528.686	1528.686

Supplementary Table 4. Sensitivity analysis (8 months margin)

	8 months margin			
	Model 0: Empty model	Model 1: Empty random effect model	Model 2: Intermediate modal	Model 3: Full model
Level 1 - screening rounds, OR (95% CI)				
N of screening round	-	-	***	***
1st round	-	-	0.156 (0.061 to 0.400)***	0.153 (0.061 to 0.380)***
2nd round	-	-	Ref	Ref
3rd to 6th rounds	-	-	0.260 (0.135 to 0.500)***	0.512 (0.276 to 0.948)*
Year of the screening round	-	-	***	
2015 to 2017	-	-	Ref	Ref
2018 to 2020	-	-	4.095 (2.056 to 8.040)***	2.603 (1.360 to 4.984)**
Age at the screening round	-	-		
<50 years	-	-	Ref	Ref
>=50 years	-	-	0.366 (0.109 to 1.233)	0.524 (0.162 to 1.688)
Number of past recall exams	-	-		
None	-	-	Ref	Ref
1 or more	-	-	1.361 (0.688 to 2.693)	1.438 (0.746 to 2.775)
Level 2 - Women				
Fixed effect, OR (95% CI)				
Patient Total N of screening rounds	-	-	-	
1 to 4 rounds	-	-	-	Ref
5 to 6 rounds	-	-	-	0.083 (0.035 to 0.196)***
Mutation status	-	-	-	
BRCA1	-	-	-	Ref
BRCA2	-	-	-	0.959 (0.488 to 1.884)
V. High Risk w/o *BRCA1* or *2*	-	-	-	0.582 (0.294 to 1.152)
Birth cohort	-	-	-	
Before 1970	-	-	-	Ref
1970-1989	-	-	-	0.688 (0.251 to 1.882)
Prevalent BC status	-	-	-	
Without	-	-	-	Ref
With	-	-	-	1.045 (0.584 to 1.868)
Standard of living of the town/city	-	-	-	
Low (Median<20479€)	-	-	-	Ref
High (Median>=20479€)	-	-	-	0.939 (0.546 to 1.616)
APL-GP of the town/city	-	-	-	
Low APL-GP (<3.7)	-	-	-	Ref
High APL-GP (>=3.7)	-	-	-	1.79 (1.044 to 3.069)
N of MRI/200 k hab. in the county	-	-	-	
<1.9	-	-	-	Ref
>=1.9	-	-	-	1.212 (0.710 to 2.069)
Random effect				
Variance of random intercept, (95% CI)	-	0.659 (0.056 to 7.690)	0.92 (0.110 to 7.677)	0.168 (0.000 to 1936.6)
Random slope	-	Age at screening round, centered		
Variance of Random slope, (95% CI)	-	0.006 (0.002 to 0.020)	0.007 (0.002 to 0.025)	0.018 (0.006 to 0.052)
VPC of Random effect	-	0.167 (0.017 to 0.700)	0.219 (0.032 to 0.700)	0.049 (4.45e-06 to 0.998)
Bayesian Information Criterion (BIC)	734.252	746.993	732.173	737.574
